# Profiles of circumscribed interests in autistic youth

**DOI:** 10.3389/fnbeh.2023.1037967

**Published:** 2023-02-09

**Authors:** Emily Spackman, Luke D. Smillie, Thomas W. Frazier, Antonio Y. Hardan, Gail A. Alvares, Andrew Whitehouse, Mirko Uljarević

**Affiliations:** ^1^Melbourne School of Psychological Sciences, University of Melbourne, Melbourne, VIC, Australia; ^2^John Carroll University, University Heights, OH, United States; ^3^Psychiatry and Behavioral Sciences, Stanford Medicine, Stanford University, Stanford, CA, United States; ^4^UWA Centre for Child Health Research, University of Western Australia, Perth, WA, Australia

**Keywords:** autism, circumscribed interests, restricted interests, unusual interests, subtyping, latent profile analysis, LPA

## Abstract

Circumscribed interests (CI) encompass a range of different interests and related behaviors that can be characterized by either a high intensity but otherwise usual topic [referred to as restricted interests (RI)] or by a focus on topics that are not salient outside of autism [referred to as unusual interests (UI)]. Previous research has suggested that there is pronounced variability across individuals in terms of the endorsement of different interests, however, this variability has not been quantified using formal subtyping approaches. Therefore, using Latent Profile Analysis in a sample of 1,892 autistic youth (M_age_ = 10.82, SD_age_ = 4.14; 420 females), this study aimed to identify subgroups based on the RU and UI profiles. Three profiles of autistic individuals were identified. They were characterized as Low CI, Predominantly RI, and Predominantly UI. Importantly, profiles differed on several key demographic and clinical variables, including age, sex composition, IQ, language level, social and communication abilities, anxiety, and obsessive-compulsive behaviors. Although replication across other samples is needed, the profiles identified in this study are potentially promising for future research given their distinct profiles of RI and UI and unique patterns of associations with key cognitive and clinical variables. Therefore, this study represents an important initial step towards more individualized assessment and support for diverse presentations of CI in autistic youth.

## Introduction

Circumscribed interests (CI) are a core subdomain of restricted and repetitive behaviors (RRB) and an important feature for the diagnosis of autism spectrum disorder (ASD; hereafter referred to as autism; American Psychiatric Association, 2013). The CI domain encompasses a range of different interests and related behaviors ranging from an intense interest in video games or music to a fascination with telephone poles or dates. Recent evidence suggests that the CI domain may be multifaceted, potentially comprising of a restricted interests (RI) subdomain that encompasses interests that, although common in terms of their content (i.e., interest in movies or animals) are characterized by unusually high intensity and an unusual interests (UI) subdomain that encompasses interests that are generally not salient or pursued outside of autism (e.g., an interest in traffic lights or timetables and dates; Uljarević et al., 2021a, b, 2022). Previous literature has demonstrated that although interests that can be classified as RI and UI are highly frequent at the group level, endorsement and specific profiles of different interests vary significantly across individuals (Klin et al., 2007; Anthony et al., 2013). Additionally, subtyping approaches have been used successfully to capture and characterize heterogeneity across both core and co-occurring aspects of the autism phenotype (Uljarević et al., 2016, 2020; Tillmann et al., 2020; Spackman et al., 2022a), including the broader RRB domain (Zheng et al., 2019). However, no research to date has attempted to explore whether there are distinct individual-level phenotypic patterns of CI.

Previous studies suggest that upwards of 75% of autistic youth experience CI and that presentations of these behaviors vary significantly across individuals (Klin et al., 2007; Anthony et al., 2013; Uljarević et al., 2021a; Spackman et al., 2022b). Using an informant-report measure of CI, Anthony et al. (2013) observed that autistic youth displayed an average of 11–15 CI, with the most common interests in autistic boys being video games and Lego and the most common interests in autistic girls being math and music. However, despite increasing insights into the presentation of CI in autism (Klin et al., 2007; Anthony et al., 2013; Grove et al., 2018; Uljarević et al., 2021a), our understanding of how these heterogenous symptoms relate to other aspects of the autism phenotype and demographic and cognitive factors remains limited.

The literature to date has reported largely inconsistent findings regarding the relationship between CI and key correlates such as age, sex, IQ, language level, social abilities, and anxiety (South et al., 2005; Klin et al., 2007; Turner-Brown et al., 2011; Anthony et al., 2013; Frazier et al., 2014; Sutherland et al., 2017; Uljarević et al., 2021a, c; Spackman et al., 2022b). This inconsistency may be in large part due to the fact that the majority of previous studies have operationalized CI as a unidimensional domain. Indeed, there is mounting evidence that restricted and unusual interests represent two distinct subdomains that show different patterns of association with key demographic, cognitive, and clinical variables including age, sex, IQ, and core autism characteristics (Uljarević et al., 2021a, 2022). Further, in addition to utilizing broad measures of CI, the above studies have focused on variable-centered approaches, meaning the relationships between these variables were assessed at a group-trend level.

The use of variable-centered approaches in heterogenous samples can potentially obscure heterogeneity and limit understanding of the factors associated with phenotypic heterogeneity (Rosato and Baer, 2012) Indeed, the cognitive and neurobiological processes underlying autism characteristics are complex and likely vary significantly between individuals showing distinct phenotypic profiles. Thus, it is important to go beyond simple case-control designs and the use of variable-centered analytical methods to gain greater insight into the mechanisms underpinning individual differences. Therefore, person-centered approaches that aim to characterize heterogeneity in a population by identifying homogeneous subgroups of similar individuals (Nylund-Gibson and Choi, 2018) are becoming increasingly utilized in autism research and have demonstrated promise when applied across different aspects of the autistic phenotype including, anxiety (Spackman et al., 2022a), social functioning (Livingston et al., 2019; Kang et al., 2020; Uljarević et al., 2020), sensory features (Ausderau et al., 2016; Uljarević et al., 2016; Tillmann et al., 2020), and RRB (Zheng et al., 2019). Within the CI domain, although studies have suggested that there is pronounced variability across individuals in terms of the endorsement of different interests, with some individuals presenting with a more constrained profile (i.e., endorsing only 1–2 interests), and others having a broader range of interests, this variability hasn’t been quantified in current literature. Further, it is not currently clear whether certain individuals tend to endorse only thoughts/behaviors that fall under the umbrella of RI, as opposed to UI, and *vice versa*. Therefore, adopting a person-centered approach to characterize CI heterogeneity, this study aimed to, firstly, identify subgroups of autistic children and adolescents based on their unique profile of RI and UI endorsement and, secondly, to characterize the association between identified CI profiles and key clinical and developmental features that have been linked to CI in previous literature, including other core characteristics of autism as well as age, sex, cognitive functioning, language levels, and anxiety.

## Methods

### Participants

The sample comprised of 1,892 autistic youth (M_age_ = 10.82, SD_age_ = 4.14; range: 3–18 years; 420 females) recruited through the Simons Foundation Powering Autism Research for Knowledge (SPARK) project. In line with previous SPARK studies (Fombonne et al., 2020; Uljarević et al., 2022), only individuals with a parent-reported professional diagnosis of ASD who scored above the cut-off (≥15) on the Social Communication Questionnaire (SCQ) were included.

### Measures

Participants completed a comprehensive online survey consisting of several measures of clinical symptoms including measures of core autism symptom severity [Social Communication Questionnaire (SCQ; Rutter et al., 2003)], anxiety (Parent-Rated Anxiety Scale—ASD [PRAS-ASD; Scahill et al., 2019], behavioral problems (Strengths and Difficulties Questionnaire [SDQ; Goodman, 2001], and sensory features [Sensory Experiences Questionnaire-3 (SEQ-3; Ausderau et al., 2014)]. Cognitive test scores, as reported by parents, were collected. This data was available for 958 participants and was coded on 10 levels (IQ = ≤ 24, 25–39, 40–54, 55–69, 70–79, 80–89, 90–109, 110–119, 120–129, and ≥ 130), which has been shown to be a sound measure of IQ (Fombonne et al., 2022). There were no significant differences in sex distribution between individuals with and without cognitive test scores.

#### Stanford dimensional assessment of repetitive behaviors (DARB)

CI was assessed using the restricted interests (15 items) and unusual interests (8 items) subscales of the Dimensional Assessment of Restricted and Repetitive Behaviors (DARB; Uljarević et al., 2022). DARB is a recently developed instrument to assess RRB and includes eight domains: repetitive sensory-motor behaviors (RSMB), insistence on sameness (IS), self-injurious behaviors (SIB), sensory sensitivity (SS), obsessions and compulsions (OC), repetitive language (RL), and two CI-related, restricted interests (RI), and unusual interests (UI). The RI and UI subscales consist of a pre-determined list of interests commonly reported in autistic youth, with a child’s interest in each topic/stimuli being rated on a five-point Likert scale from “mild-interest” to “very intense/extreme interest.” Specific interests were chosen based on the systematic search of the literature to ensure comprehensive coverage of relevant RI and UI. In addition, the DARB also includes an open-ended item for parents to list any additional interests not included in the questionnaire. Given that the majority of parents reported that all interests were captured by the pre-determined list and that they did not quantify reported interests using Likert scale, open ended responses were not used in the current study. RI and UI have been found to have good internal consistency (omega values: RI = 0.76; UI = 0.78) and excellent test-retest stability (Intraclass correlation coefficients: RI = 0.90; UI = 0.90). The remaining six subscales of the DARB we used to explore the relationships between presentations and other RRBs.

### Statistical analysis

Latent Profile Analysis (LPA), using the maximum likelihood estimation method (Morin et al., 2011), was conducted to explore profiles of response patterns on all CI items across the two DARB subscales. Although no prior studies have attempted to identify latent profiles in CI presentation, previous subtyping studies examining both the core and co-occurring symptoms of autism have yielded up to six subgroups (Agelink van Rentergem et al., 2021). To ensure that any relevant profiles were captured, models with 1–6 profiles were estimated. The final model was selected by considering the combination of the following fit indices: (i) the Akaïke Information Criterion (AIC); (ii) the Bayesian Information Criterion (BIC); (iii) the sample-size Adjusted BIC (ABIC); (iv) the Bootstrap Likelihood Ratio Test (BLRT); (v) Vuong–Lo–Mendell–Rubin likelihood ratio test (VLMR); and (vi) entropy values (Morin et al., 2011; Nylund-Gibson and Choi, 2018). A better absolute fit was indicated by lower AIC, BIC, and ABIC values and higher entropy. The VLMR and BLRT are relative fit indices that assess fit improvement with the addition of each subgroup (k vs. k–1; Nylund-Gibson and Choi, 2018). In addition, model selection was also guided by parsimony and interpretability.

Once the profiles were extracted, subgroup-level differences in mean RI and UI item scores, as well as differences in clinical features including age, sex, IQ, language levels, social abilities, anxiety, and other RRBs, were examined *via* one-way analysis of variance (ANOVA). Bootstrapping using 5,000 resamples was conducted to provide more robust statistics and account for the potential skewness of the data (Efron and Tibshirani, 1993). Omega squared (ω^2^) and Cohen’s d was computed as a measure of effect size for relevant comparisons. The alpha criterion was adjusted using the Benjamini et al. (2001) method to control the false discovery rate for multiple ANOVAs and a Bonferroni correction was applied to *post hoc* comparisons. All analyses were conducted in SPSS version 28.0 (IBM Corp, 2021), with the exception of the LPA which were conducted in MPlus 8.2 (Muthén and Muthén, 1998–2017).

## Results

Descriptive statistics are presented in Table 1. Fit statistics for the 1–6 profile solutions are presented in Table 2. As can be seen, the information criterion values (AIC, BIC, and Adjusted-BIC) continued to decrease incrementally with each added profile, indicating a better fit, however, reductions were small after the addition of the third profile. Additionally, the BLRT and VLMR were no longer significant by the fourth profile, indicating no significant improvement with the addition of a fourth profile. The three-profile solution exhibited high entropy (0.92) and high latent profile probabilities (Profile 1 = 0.97, 2 = 0.93, 3 = 1.00), indicating good classification accuracy (Nylund-Gibson and Choi, 2018). Examination of the adjacent two- and four-profile solutions confirmed the added value of the third profile when compared to the two-profile solution, as the two-profile solution fell along a severity gradient, whereas the three-profile solution formed groups that differed meaningfully between items under the RI and UI domains. However, the addition of the fourth profile did not yield an additional profile with distinct RI and UI presentation. Therefore, the three-profile solution was selected as the optimal solution.

**Table 1:** Sample demographic characteristics.

Characteristic		M (SD)	Range
Age		10.82 (4.14)	3–18
Cognitive functioning		6.30 (2.53)	1–10
Characteristic			%
Sex			
Male			76.6
Female			22.4
Race and ethnicity			
African American			8.0
Asian			5.8
Hispanic			7.4
Native American/Native Hawaiian			2.2
White			70.3
Mixed race			6.3
Intellectual disability			24.8

Note. Cognitive functioning was measured using 10 levels (IQ = ≤24, 25–39, 40–54, 55–69, 70–79, 80–89, 90–109, 110–119, 120–129, and ≥130).

**Table 2:** Latent profile analysis fit statistics across models.

# of classes	LL	Nfp	AIC	BIC	Adjusted BIC	Entropy	VLMR *p*-value	BLRT *p*-value
1	−63,355.6	46	126,803.1	127,057.9	126,911.8	-	-	-
2	−61,248.2	70	122,636.3	123,024	122,801.7	0.901	<0.001	<0.001
3	−60,209.6	94	120,607.3	121,127.9	120,829.3	0.916	<0.001	<0.001
4	−59,527.6	118	119,291.2	119,944.8	119,569.9	0.923	0.3449	0.347
5	−58,891.5	142	118,067	118,853.4	118,402.3	0.937	0.0295	0.030
6	−58,151.6	166	116,635.2	117,555.8	117,028.4	0.927	0.3049	0.304

Note. LL = log likelihood; Nfp = number of free parameters; AIC = Akaïke Information Criterion; BIC = Bayesian Information Criterion; VLMR = Vuong–Lo–Mendell–Rubin likelihood ratio test; BLRT = Bootstrap likelihood ratio test.

For each participant, the profile assignment was determined by maximum probability. Differences between profiles were significant for all CI items, except between profiles 2 and 3 for RI items “TV shows or movies”, “watching sports”, “playing sports”, “psychology” and “science”. As can be seen in Table 3, participants in profile 1 (*n* = 1,357) scored significantly lower across all CI items and the profile was, therefore, labeled “low CI”. Participants in profile 2 (*n* = 416) scored higher than profile 1, but lower than profile 3 (*n* = 106), on all UI items, but scored higher than or equal to profile 3 on all RI items except interest in vehicles. Thus, profile 2 was labeled predominantly RI. And profile 3 was predominantly UI. However, it is important to note that the difference between profiles 2 and 3 was much greater on UI items than RI items, meaning that the predominantly UI group still presented with elevated RI (see Table 3).

**Table 3:** LPA profile differences on RI and UI items.

				Profile 1	Profile 2	Profile 3	*Post hoc* comparisons		
	*F*	*p*	ω^2^	M	M	M	1v2	1v3	2v3
				(SD)	(SD)	(SD)			
Unusual interests									
Number	3,437.25	<0.001	0.786	0.04 (0.20)	0.18 (0.39)	2.64 (0.84)	<0.001	<0.001	<0.001
Dates/times	149.48	<0.001	0.138	0.21 (0.64)	0.70 (1.16)	1.42 (1.37)	<0.001	<0.001	<0.001
Unusual items	104.23	<0.001	0.100	0.31 (0.77)	0.85 (1.22)	1.33 (1.51)	<0.001	<0.001	<0.001
Favorite person	239.13	<0.001	0.203	0.63 (0.96)	1.72 (1.38)	2.32 (1.38)	<0.001	<0.001	<0.001
Collects unusual items	191.97	<0.001	0.170	0.57 (0.96)	1.60 (1.46)	2.06 (1.49)	<0.001	<0.001	<0.001
Categorization	184.41	<0.001	0.165	0.10 (0.39)	0.57 (0.93)	0.99 (1.17)	<0.001	<0.001	<0.001
Visual aspects of objects	196.60	<0.001	0.174	0.58 (0.95)	1.68 (1.42)	1.96 (1.42)	<0.001	<0.001	0.007
Specific objects	143.49	<0.001	0.141	0.79 (1.06)	1.76 (1.46)	2.10 (1.45)	<0.001	<0.001	0.034
Restricted interests									
History	160.35	<0.001	0.145	0.34 (0.73)	1.27 (1.32)	0.66 (1.12)	<0.001	0.001	<0.001
Listening to music	110.70	<0.001	0.105	1.49 (1.19)	2.72 (2.03)	1.84 (1.96)	<0.001	0.003	<0.001
Practicing arts	138.43	<0.001	0.128	0.94 (1.08)	2.02 (1.38)	1.51 (1.49)	<0.001	<0.001	<0.001
Technology	95.28	<0.001	0.091	1.86 (1.39)	2.91 (1.20)	2.40 (1.41)	<0.001	<0.001	0.005
Mythology	232.90	<0.001	0.198	0.21 (0.58)	1.20 (1.29)	0.73 (1.15)	<0.001	<0.001	<0.001
Fictional characters	193.41	<0.001	0.170	1.32 (1.22)	2.65 (1.24)	2.16 (1.41)	<0.001	<0.001	0.010
Literature	210.95	<0.001	0.183	0.37 (0.69)	1.35 (1.21)	0.96 (1.19)	<0.001	<0.001	<0.001
Animals/nature	198.32	<0.001	0.174	1.22 (1.12)	2.52 (1.26)	2.11 (1.51)	<0.001	<0.001	0.004
Science	153.07	<0.001	0.140	0.69 (0.97)	1.72 (1.30)	1.38 (1.39)	<0.001	<0.001	0.067
Famous people	159.15	<0.001	0.144	0.21 (0.56)	0.99 (1.22)	0.78 (1.24)	<0.001	<0.001	0.049
TV shows/movies	148.95	<0.001	0.136	1.43 (1.16)	2.49 (1.23)	2.36 (1.41)	<0.001	<0.001	1.00
Playing sports	32.00	<0.001	0.032	0.37 (0.74)	0.71 (0.97)	0.69 (1.01)	<0.001	<0.001	1.00
Psychology	96.92	<0.001	0.093	0.07 (0.35)	0.50 (0.94)	0.56 (1.12)	<0.001	<0.001	1.00
Watching sports	49.82	<0.001	0.049	0.23 (0.66)	0.58 (1.00)	0.77 (1.15)	<0.001	<0.001	0.314
Vehicles	50.26	<0.001	0.050	0.95 (1.24)	1.54 (1.48)	1.86 (1.43)	<0.001	<0.001	0.012

Note. All ANOVA *p*-values remained significant after FDR correction was applied.

Table 4 shows age, IQ, social abilities, anxiety, behavior problems, sensory features, and other RRB comparisons across the three CI profiles identified in LPA. Participants in the predominantly RI profile were significantly older (*F* = 9.21, *p* < 0.001, ω^2^ = 0.009) scored higher on cognitive functioning (*F* = 10.41, *p* < 0.001, ω^2^ = 0.019), and showed higher language levels ((*F* = 27.84, *p* < 0.001, ω^2^ = 0.028) than the participants in low CI profile or predominantly UI profile. They also presented with more anxiety (*F* = 5.64, *p* = 0. 004, ω^2^ = 0.007) and obsessive/compulsive behaviors (*F* = 4.87, *p* = 0.008, ω^2^ = 0.004) than the low CI profile but did not differ significantly on either anxiety or Obsessive-Compulsive Behaviors from the predominantly UI profile. Finally, a chi-square analysis revealed that sex distributions differed significantly across profiles (χ^2^ = 9.09, *p* = 0.011), however, the effect size for this difference was small (*V* = 0.070). Post hoc analysis with a Bonferroni correction revealed that females were more likely than males to be in the Predominantly RI profile, while males were more likely than females to be in the low CI profile. The sex composition on the Predominantly UI profile did not differ significantly.

**Table 4:** Profile differences across demographic, cognitive, and clinical characteristics.

	F	p-value	ω^2^	Profile 1	Profile 2	Profile 3	Post hoc comparisons		
				M	M	M	1v2	1v3	2v3
				(SD)	(SD)	(SD)		
Age	9.21	<0.001*	0.009	10.62 (4.19)	11.54 (3.99)	10.05 (4.01)	<0.001	0.462	0.003
Cognitive Functioning^1^	10.41	<0.001*	0.019	6.11 (2.50)	6.96 (2.42)	5.96 (2.93)	<0.001	0.999	0.031
Social Communication	4.77	0.009*	0.004	13.95 (0.15)	13.71 (0.27)	15.54 (0.54)	0.999	0.013	0.007
Language level	27.84	<0.001*	0.028	3.40 (1.00)	3.78 (0.56)	3.38 (0.92)	<0.001	0.999	<0.001
Anxiety	5.64	0.004*	0.007	25.13 (16.09)	28.86 (16.66)	25.90 (16.33)	0.002	0.999	0.512
DARB RMSB	3.54	0.029	0.003	17.16 (13.49)	15.71 (13.35)	14.37 (12.71)	0.160	0.117	1.00
DARB SIB	2.89	0.056	0.002	3.68 (4.14)	4.25 (4.73)	3.89 (3.64)	0.050	0.999	0.999
DARB IS	0.14	0.870	−0.001	17.61 (15.13)	17.27 (16.98)	16.98 (14.43)	0.999	0.999	0.999
DARB SS	0.93	0.397	0.000	15.45 (8.87)	15.43 (9.38)	16.70 (9.46)	0.999	0.537	0.621
DARB RL	0.21	0.807	−0.001	14.13 (10.68)	14.43 (10.48)	14.64 (11.00)	0.999	0.999	0.999
DARB OCB	4.87	0.008*	0.004	3.92 (5.28)	4.84 (6.67)	4.80 (6.07)	0.012	0.371	0.999

Note: *, significant after FDR correction applied; SIB, self-injurious behaviors; IS, insistence on sameness; SS, sensory sensitivity; RL, Repetitive Language; OCB, Obsessive-Compulsive Behaviors; ^1^Cognitive functioning was measured using 10 levels (IQ = ≤24, 25−39, 40−54, 55−69, 70−79, 80−89, 90−109, 110−119, 120−129, and ≥130); Higher social/communication score reflects greater impairment.

## Discussion

Previous research has suggested that CI is a broad domain encompassing a range of different interests and related behaviors that can be classified as either Restricted Interests (RI) or Unusual Interests (UI). However, no study to date has provided a systematic characterization of whether different autistic individuals show distinct CI profiles across the RI and UI subdomains. Given that previous literature has suggested that different manifestations of CI may show unique patterns of association with clinical features (Uljarević et al., 2021a, 2022), characterizing profiles of distinct aspects of CI, including RI and UI, among individuals with autism, is an important step toward better understanding these complex and heterogenous domains. In the current study, three profiles were identified, labeled as low CI, predominantly RI, and predominantly UI based on the constellation of specific CI. Identified profiles differed significantly on age, sex composition, IQ, language levels, social characteristics, anxiety, and obsessive-compulsive behaviors, which provides important, albeit preliminary, evidence that different profiles of CI presentations may be underpinned by distinct cognitive and potentially even neurobiological mechanisms.

The findings that participants in the predominantly RI profile were significantly older and demonstrated higher cognitive functioning and language abilities are somewhat consistent with variable-level trends observed in a previous study that found IQ and age were positive predictors of RI and negative predictors of UI (Uljarević et al., 2022). Whilst other variable-level studies have reported either no relationship between developmental variables and CI (Anthony et al., 2013), or a reverse relationship for IQ (Uljarević et al., 2021c), these studies have typically excluded individuals with a co-occurring intellectual disability or have used broad measures of CI. Interestingly, it has been reported that autistic individuals with delayed speech onset provide more perceptual descriptions of their interests (i.e., related to color, appearance, order, etc.), whereas autistic individuals without delayed speech onset provide more thematic descriptions of their interests (i.e., related to knowledge or semantics; Chiodo et al., 2017). Therefore, it is possible that individuals in the predominantly UI profile presenting with lower language and developmental levels may be linked to UI having a more perceptual focus than RI, which instead, may be more thematic. Alternatively, another explanation for the current finding that participants in the predominantly RI profile scored higher on development-related variables may be that many RI (e.g., science and technology) require a certain level of cognitive ability; thus, it is possible that younger children and individuals with a co-occurring ID may be less likely to pursue these interests. Further, it is also possible that these profiles may show different patterns of trajectory across ages and developmental levels, something that future longitudinal studies may wish to explore.

The predominantly RI profile demonstrated higher anxiety and compulsive behaviors than the predominantly UI profile. However, neither of these profiles differed significantly from the low CI profile on these variables. Spiker et al. (2012) suggested that engaging in CI may serve as a means to reduce anxiety and therefore CI may be higher in individuals who experience elevated anxiety. Whilst the current finding that neither the predominantly RI nor UI profiles differed from the low CI profile on anxiety scores is not directly consistent with this suggestion, the relationship between anxiety and CI is not well explored in the current literature. It is also possible that the difference in anxiety between RI and UI profiles identified in this study could be driven by a common underpinning mechanism. Therefore, future studies may wish to explore other potential third variables, such as executive functioning or self-regulation, both of which have been linked to anxiety and CI (Anthony et al., 2013; Hollocks et al., 2014; Demetriou et al., 2019; Faja and Nelson Darling, 2019).

Importantly, the predominantly UI profile demonstrated significantly greater social and communication impairments than the other two profiles. Although previous studies have reported that higher CI is associated with greater social impairments (Klin et al., 2007; Clements et al., 2018; Carter et al., 2020; Uljarević et al., 2021c), these studies treated CI as a unidimensional construct. The current study, with its large sample size, is the first investigation to identify a cluster of participants presenting with higher UI and moderate RI that displayed more severe social impairments than individuals in other profiles. This finding builds on previous evidence that RI and UI demonstrate different variable-level associations with social and communication characteristics, such that higher social abilities predicted higher RI and lower UI (Uljarević et al., 2022). Further, previous literature has suggested that despite being associated with more social impairments, CI can also have adaptive benefits for promoting socialization when a conversational partner who shares their interest can be found (Boyd et al., 2007; Harrop et al., 2019; Smerbeck, 2019). Whether current findings reflect the fact that this adaptive function may apply more strongly to RI than UI, or whether individuals with higher social abilities, to begin with, may be more likely to develop an interest in RI topics over UI is currently unclear and warrants further investigation.

Finally, the three profiles demonstrated different sex compositions with males significantly more likely to be in the low CI profile and females significantly more likely to be in the predominantly RI profile. Although it is difficult to situate findings in the context of previous literature as this is the first study to capture subgroups based on CI patterns, that males were more likely to be in the low CI profile is somewhat inconsistent with previous studies that have reported no sex-based differences in the intensity of CI (Anthony et al., 2013; Sutherland et al., 2017; Spackman et al., 2022b). However, the finding that females were more likely than males to be in the Predominantly RI profile, is broadly consistent with previous literature that suggests the interests of autistic females more closely align with the interests of neurotypical females than autistic males (Harrop et al., 2018; Bourson and Prevost, 2022). Further, by identifying a profile of interests, mostly consisting of RI, that females are more likely to display, this study builds on previous group-level findings using the DARB that reported several specific RI items were more commonly endorsed by females than males—including having a favorite person or an interest in music, art, literature, and psychology (Spackman et al., 2022b). In summary, the use of person-centered analysis in the current study has identified clusters of participants that were further differentiated by key demographic, cognitive, and clinical validators, thus providing important, albeit preliminary, evidence that different profiles of CI presentations may be underpinned by distinct cognitive mechanisms.

The present findings should be considered in light of several limitations. First, as data was collected through SPARK, it was not possible to independently confirm ASD diagnoses. However, previous studies exploring this limitation of SPARK have found a high validity of ASD diagnoses (Feliciano et al., 2018; Fombonne et al., 2020, 2022) and the current study included only participants scoring above the SCQ cut-off score. Second, given the challenges of measuring autism characteristics in non-verbal individuals, the current study relied exclusively on parent-report measures. Whilst this has shown to be an efficient means of measuring autism characteristics in samples including non-verbal participants (Irwin et al., 2012), we acknowledge the importance of multi-informant methods in autism research (Stratis and Lecavalier, 2015). Future research may wish to expand on these findings using self-report measures and consider additional features on which profiles might differ, including different facets of executive functioning and self-regulation. Further, as the field moves towards more transdiagnostic understandings of neurodevelopment, it will be important for future studies to explore whether identified profiles might also be replicated in other neurodevelopmental conditions and how they compare with profiles that might be observed in typically developing children. Finally, it has been suggested that phenotypic heterogeneity in autism likely reflects diverse biological etiologies. It is thus important for future studies to explore whether distinct RI- and UI-based profiles are underpinned by specific neurobiological signatures. In particular, it will be important to focus on structural and functional atypicalities in cognitive and limbic cortico-striatal circuits that have been linked to specific aspects of executive functioning and reward processing and have been suggested to potentially underpin different aspects of CI (Langen et al., 2011; Carter et al., 2020). Therefore, although significantly more research is needed to determine the generalizability of current profiles, identifying subgroups of participants based on distinct presentations of precisely defined core autism characteristics and underlying cognitive processes may represent an important step toward elucidating the complexities of these brain-behavior relationships.

In summary, three profiles of CI presentations were identified that differed in key clinical features: low CI, predominantly UI, and predominantly RI. Although derived profiles are promising and potentially informative, significantly more research is needed to replicate and explore the utility of these subtypes for understanding neurobiological underpinnings, prognostic implications, and therapeutic relevance. Additional research will be important to characterize potential mechanisms underlying distinct presentations, in particular focusing on executive functioning and reward processing, and their relevance for informing clinical management and support strategies.

## Data availability statement

The original contributions presented in the study are included in the article, further inquiries can be directed to the corresponding author.

## Ethics statement

The studies involving human participants were reviewed and approved by Stanford University Institutional Review Board (IRB #53669). The patients/participants provided their written informed consent to participate in this study.

## Author contributions

ES and MU designed the study with input from LS, AH, TF, GA, and AW. MU, AH, and TF collected the data. ES, MU, AH, and TF had full access to the data and ES and MU conducted the analysis. ES, MU, and LS drafted the initial manuscript. All authors contributed to the article and approved the submitted version.

## References

[B1] Agelink van Rentergem J. A. Deserno M. K. Geurts H. M. (2021). Validation strategies for subtypes in psychiatry: a systematic review of research on autism spectrum disorder. Clin. Psychol. Rev. 87:102033. 10.1016/j.cpr.2021.102033 33962352

[B2] American Psychiatric Association (2013). Diagnostic and Statistical Manual of Mental Disorders (5th ed.). Washington, DC: American Psychiatric Association.

[B3] Anthony L. G. Kenworthy L. Yerys B. E. Jankowski K. F. James J. D. Harms M. B.. (2013). Interests in high-functioning autism are more intense, interfering and idiosyncratic than those in neurotypical development. Dev. Psychopathol. 25, 643–652. 10.1017/S0954579413000072 23880382 PMC4543385

[B5] Ausderau K. Sideris J. Furlong M. Little L. M. Bulluck J. Baranek G. T. (2014). National survey of sensory features in children with ASD: factor structure of the sensory experience questionnaire (3.0). J. Autism Dev. Disord. 44, 915–925. 10.1007/s10803-013-1945-1 24097141 PMC3949144

[B4] Ausderau K. K. Sideris J. Little L. M. Furlong M. Bulluck J. C. Baranek G. T. (2016). Sensory subtypes and associated outcomes in children with autism spectrum disorders. Autism Res. 9, 1316–1327. 10.1002/aur.1626 27135214

[B6] Benjamini Y. Drai D. Elmer G. Kafkafi N. Golani I. (2001). Controlling the false discovery rate in behavior genetics research. Behav. Brain Res. 125, 279–284. 10.1016/s0166-4328(01)00297-2 11682119

[B7] Bourson L. Prevost C. (2022). Characteristics of restricted interests in girls with ASD compared to boys: a systematic review of the literature. Eur. Child Adolesc. Psychiatry 10.1007/s00787-022-01998-5[Online ahead of print]. 35644857

[B8] Boyd B. A. Conroy M. A. Mancil G. R. Nakao T. Alter P. J. (2007). Effects of circumscribed interests on the social behaviors of children with autism spectrum disorders. J. Autism Dev. Disord. 37, 1550–1561. 10.1007/s10803-006-0286-8 17146704

[B9] Carter R. M. Jung H. Reaven J. Blakeley-Smith A. Dichter G. S. (2020). A nexus model of restricted interests in autism spectrum disorder. Front. Hum. Neurosci. 14:212. 10.3389/fnhum.2020.00212 32581753 PMC7283772

[B200] Chiodo L. Majerus S. Mottron L. (2017). Typical versus delayed speech onset influences verbal reporting of autistic interests. Mol. Autism 8:35. 10.1186/s13229-017-0155-7 28736607 PMC5520365

[B10] Clements C. C. Zoltowski A. R. Yankowitz L. D. Yerys B. E. Schultz R. T. Herrington J. D. (2018). Evaluation of the social motivation hypothesis of autism: a systematic review and meta-analysis. JAMA Psychiatry 75, 797–808. 10.1001/jamapsychiatry.2018.1100 29898209 PMC6143096

[B11] Demetriou E. A. DeMayo M. M. Guastella A. J. (2019). Executive function in autism spectrum disorder: history, theoretical models, empirical findings and potential as an endophenotype. Front. Psychiatry 10:753. 10.3389/fpsyt.2019.00753 31780959 PMC6859507

[B12] Efron B. Tibshirani R. J. (1993). An Introduction to the Bootstrap. New York, NY: Chapman and Hall.

[B13] Faja S. Nelson Darling L. (2019). Variation in restricted and repetitive behaviors and interests relates to inhibitory control and shifting in children with autism spectrum disorder. Autism 23, 1262–1272. 10.1177/1362361318804192 30394786 PMC6499722

[B14] Feliciano P. Daniels A. M. Snyder L. G. Beaumont A. Camba A. Esler A.. (2018). SPARK: a US cohort of 50,000 families to accelerate autism research. Neuron 97, 488–493. 10.1016/j.neuron.2018.01.015 29420931 PMC7444276

[B100] Fombonne E. Green Snyder L. Daniels A. Feliciano P. Chung W. SPARK Consortium (2020). Psychiatric and medical profiles of autistic adults in the SPARK cohort. J. Autism Dev. Disord. 50, 3679–3698. 10.1007/s10803-020-04414-6 32096123

[B101] Fombonne E. Coppola L. Mastel S. O’Roak B. J. (2022). Validation of autism diagnosis and clinical data in the SPARK cohort. J. Autism Dev. Disord. 52, 3383–3398. 10.1007/s10803-021-05218-y 34328611

[B17] Frazier T. W. Georgiades S. Bishop S. L. Hardan A. Y. (2014). Behavioral and cognitive characteristics of females and males with autism in the Simons Simplex Collection. J. Am. Acad. Child Adolesc. Psychiatry 53, 329–340.e1-3. 10.1016/j.jaac.2013.12.004 24565360 PMC3935179

[B18] Goodman R. (2001). Psychometric properties of the strengths and difficulties questionnaire. J. Am. Acad. Child Adolesc. Psychiatry 40, 1337–1345. 10.1097/00004583-200111000-00015 11699809

[B19] Grove R. Hoekstra R. A. Wierda M. Begeer S. (2018). Special interests and subjective wellbeing in autistic adults. Autism Res. 11, 766–775. 10.1002/aur.1931 29427546

[B21] Harrop C. Amsbary J. Towner-Wright S. Reichow B. Boyd B. A. (2019). That’s what I like: the use of circumscribed interests within interventions for individuals with autism spectrum disorder. a systematic review. Res. Autism Spectrum Disord. 57, 63–86. 10.1016/j.rasd.2018.09.008

[B20] Harrop C. Jones D. Zheng S. Nowell S. W. Boyd B. A. Sasson N. (2018). Sex differences in social attention in autism spectrum disorder. Autism 11, 1264–1275. 10.1002/aur.1997 30403327 PMC7468514

[B22] Hollocks M. J. Jones C. R. Pickles A. Baird G. Happé F. Charman T.. (2014). The association between social cognition and executive functioning and symptoms of anxiety and depression in adolescents with autism spectrum disorders. Autism Res. 7, 216–228. 10.1002/aur.1361 24737743

[B212] IBM Corp. (2021). IBM SPSS Statistics for Windows, Version 28.0. Armonk, NY: IBM Corp.

[B23] Irwin D. E. Gross H. E. Stucky B. D. Thissen D. DeWitt E. M. Lai J. S.. (2012). Development of six PROMIS pediatrics proxy-report item banks. Health Qual. Life Outcomes 10:22. 10.1186/1477-7525-10-22 22357192 PMC3312870

[B24] Klin A. Danovitch J. H. Merz A. B. Volkmar F. R. (2007). Circumscribed interests in higher functioning individuals with autism spectrum disorders: an exploratory study. Res. Pract. Persons Severe Disabil. 32, 89–100. 10.2511/rpsd.32.2.89

[B25] Kang E. Gadow K. D. Lerner M. D. (2020). Atypical communication characteristics, differential diagnosis and the autism spectrum disorder phenotype in youth. J. Clin. Child Adolesc. Psychol. 49, 251–263. 10.1080/15374416.2018.1539912 30644771

[B26] Langen M. Durston S. Kas M. J. van Engeland H. Staal W. G. (2011). The neurobiology of repetitive behavior: …and men. Neurosci. Biobehav. Rev. 35, 356–365. 10.1016/j.neubiorev.2010.02.005 20153769

[B27] Livingston L. A. Colvert E. Social Relationships Study Team Bolton P. Happé F. (2019). Good social skills despite poor theory of mind: exploring compensation in autism spectrum disorder. J. Child Psychol. Psychiatry 60, 102–110. 10.1111/jcpp.12886 29582425 PMC6334505

[B28] Morin A. J. S. Morizot J. Boudrias J. S. Madore I. (2011). A multifoci person-centered perspective on workplace affective commitment: a latent profile/factor mixture analysis. Organ. Res. Methods 14, 58–90. 10.1177/1094428109356476

[B102] Muthén L. K. Muthén B. O. (1998–2017). Mplus User’s Guide, (8th ed.). Los Angeles, CA: Muthén & Muthén.

[B103] Nylund-Gibson K. Choi A. Y. (2018). Ten frequently asked questions about latent class analysis. Transl. Issues Psychol. Sci. 4, 440–461. 10.1037/tps0000176

[B104] Rosato N. S. Baer J. C. (2012). Latent class analysis: a method for capturing heterogeneity. Soc. Work Res. 36, 61–69. 10.1093/swr/svs006

[B105] Rutter M. Bailey A. Lord C. (2003). The Social Communication Questionnaire. Los Angeles, CA: Western Psychological Services.

[B33] Scahill L. Lecavalier L. Schultz R. T. Evans A. N. Maddo B. (2019). Development of the parent-rated anxiety scale for youth with autism spectrum disorder. J. Am. Acad. Child Adolesc. Psychiatry 58, 887–896. 10.1016/j.jaac.2018.10.016 30797036

[B34] Smerbeck A. (2019). The survey of favorite interests and activities: assessing and understanding restricted interests in children with autism spectrum disorder. Autism 23, 247–259. 10.1177/1362361317742140 29172638

[B35] South M. Ozonoff S. McMahon W. M. (2005). Repetitive behavior profiles in Asperger syndrome and high-functioning autism. J. Autism Dev. Disord. 35, 145–158. 10.1007/s10803-004-1992-8 15909401

[B36] Spackman E. Lerh J. W. Rodgers J. Hollocks M. J. South M. McConachie H.. (2022a). Understanding the heterogeneity of anxiety in autistic youth: a person-centered approach. Autism Res. 15, 1742–1754. 10.1002/aur.2744 35642170

[B37] Spackman E. Smillie L. Frazier T. W. Hardan A. Y. Alvares G. A. Whitehouse A.. (2022b). Characterizing restricted and unusual interests in autistic youth. Autism Res. 10.1002/aur.2863[Online ahead of print]. 36453155

[B38] Spiker M. A. Lin C. E. Van Dyke M. Wood J. J. (2012). Restricted interests and anxiety in children with autism. Autism 16, 306–320. 10.1177/1362361311401763 21705474

[B39] Stratis E. A. Lecavalier L. (2015). Informant agreement for youth with autism spectrum disorder or intellectual disability: a meta-analysis. J. Autism Dev. Disord. 45, 1026–1041. 10.1007/s10803-014-2258-8 25253177

[B40] Sutherland R. Hodge A. Bruck S. Costley D. Klieve H. (2017). Parent-reported differences between school-aged girls and boys on the autism spectrum. Autism 21, 785–794. 10.1177/1362361316668653 28287270

[B41] Tillmann J. Uljarevic M. Crawley D. Loth E. Murphy D. Buitelaar J.. (2020). Dissecting the phenotypic heterogeneity in sensory features in autism spectrum disorder: a factor mixture modelling approach. Mol. Autism 11:67. 10.1186/s13229-020-00367-w 32867850 PMC7457751

[B42] Turner-Brown L. M. Lam K. S. L. Holtzclaw T. N. Dichter G. S. Bodfish J. W. (2011). Phenomenology and measurement of circumscribed interests in autism spectrum disorders. Autism 15, 437–456. 10.1177/1362361310386507 21454386 PMC3709861

[B43] Uljarević M. Alvares G. A. Steele M. Edwards J. Frazier T. W. Hardan A. Y.. (2021a). Toward better characterization of restricted and unusual interests in youth with autism. Autism 26, 1296–1304. 10.1177/13623613211056720 34818937 PMC9126999

[B44] Uljarević M. Jo B. Frazier T. W. Scahill L. Youngstrom E. A. Hardan A. Y. (2021b). Using the big data approach to clarify the structure of restricted and repetitive behaviors across the most commonly used autism spectrum disorder measures. Mol. Autism 12:39. 10.1186/s13229-021-00419-9 34044873 PMC8162018

[B46] UljarevićM.FrazierT. W.JoB.BillinghamW. D.CooperM. N.YoungstromE. A.. (2021c). Big data approach to characterize restricted and repetitive behaviors in autism. J. Am. Acad. Child Adolesc. Psychiatry 61, 446–457. 10.1016/j.jaac.2021.08.00634391858

[B45] Uljarević M. Frazier T. W. Jo B. Scahill L. Youngstrom E. A. Spackman E.. (2022). Dimensional assessment of restricted and repetitive behaviours: development and preliminary validation of a new measure. J. Am. Acad. Child Adolesc. Psychiatry 10.1016/j.jaac.2022.07.863[Online ahead of print]. 36526162

[B47] Uljarević M. Lane A. Kelly A. Leekam S. (2016). Sensory subtypes and anxiety in older children and adolescents with autism spectrum disorder. Autism Res. 9, 1073–1078. 10.1002/aur.1602 26765165

[B48] Uljarević M. Phillips J. M. Schuck R. K. Schapp S. Solomon E. M. Salzman E.. (2020). Exploring social subtypes in autism spectrum disorder: a preliminary study. Autism Res. 13, 1335–1342. 10.1002/aur.2294 32187854

[B49] Zheng L. Grove R. Valsamma E. (2019). Spectrum or subtypes? a latent profile analysis of restricted and repetitive behaviors in autism. Res. Autism Spectr. Disord. 57, 46–54. 10.1016/j.rasd.2018.10.003

